# Supplementary material for: Activating the Intrinsic Pathway of Apoptosis Using BIM BH3 Peptides Delivered by Peptide Amphiphiles with Endosomal Release
Source: Materials (Basel). 2019 Aug 12;12(16):2567. doi: 10.3390/ma12162567 (PMC6719084; doi:10.3390/ma12162567)
Supplement: Supplementary file 1 [file materials-12-02567-s001.pdf]

materials

Article

Activating the Intrinsic Pathway of Apoptosis Using BIM BH3 Peptides Delivered by Peptide Amphiphiles with Endosomal Release

Mathew R. Schnorenberg [1,2,3,‡], Joseph A. Bellairs [2,‡], Ravand Samaeekia [1,2], Handan Acar [1,2,†], Matthew V. Tirrell [1] and James L. LaBelle [2,*]

1 Pritzker School of Molecular Engineering, University of Chicago, Chicago, IL 60637, USA
2 Department of Pediatrics, Section of Hematology/Oncology, University of Chicago, Chicago, IL 60637, USA
3 Medical Scientist Training Program, University of Chicago, Chicago, IL 60637, USA
* Correspondence: jlabelle@peds.bsd.uchicago.edu
† Current address: Stephenson School of Biomedical Engineering, University of Oklahoma, Norman, OK 73071, USA
‡ These authors contributed equally to this work

Received: 29 June 2019; Accepted: 5 August 2019; Published: 12 August 2019

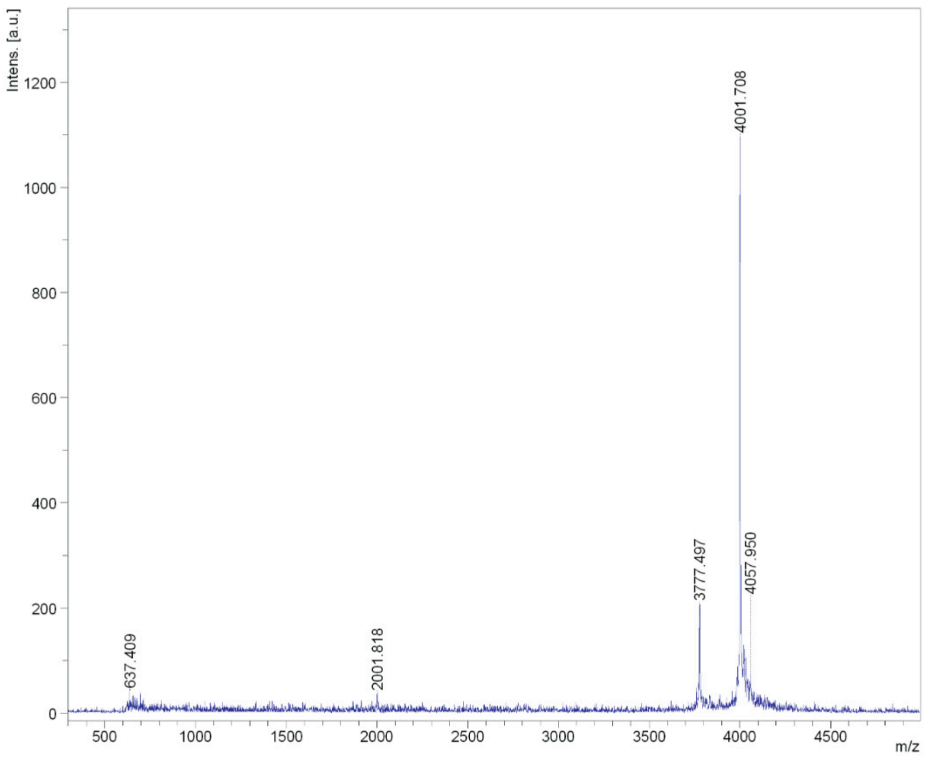

Figure S1. MALDI-TOF spectrum of $BIM_{A,K}PA_1$. Expected molecular weight is 4001 Da.

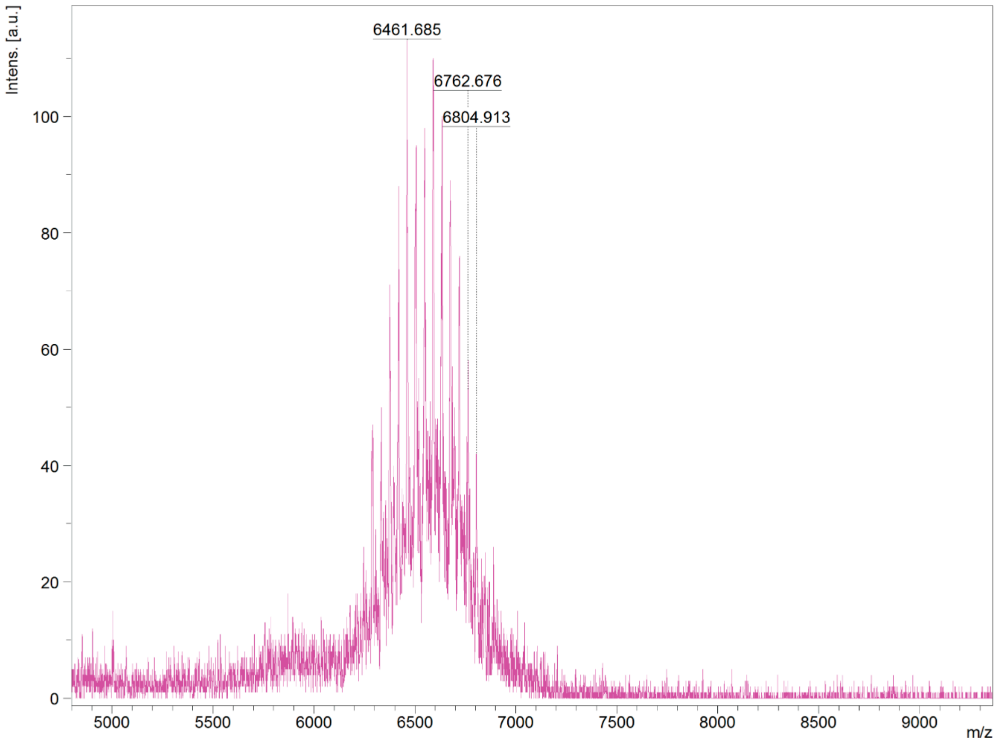

Figure S2. MALDI-TOF spectrum of $BIM_{A,cath,K}PA_2$. The expected average molecular weight is ~6452 Da, with polydispersity due to the PEG spacer in the tail.

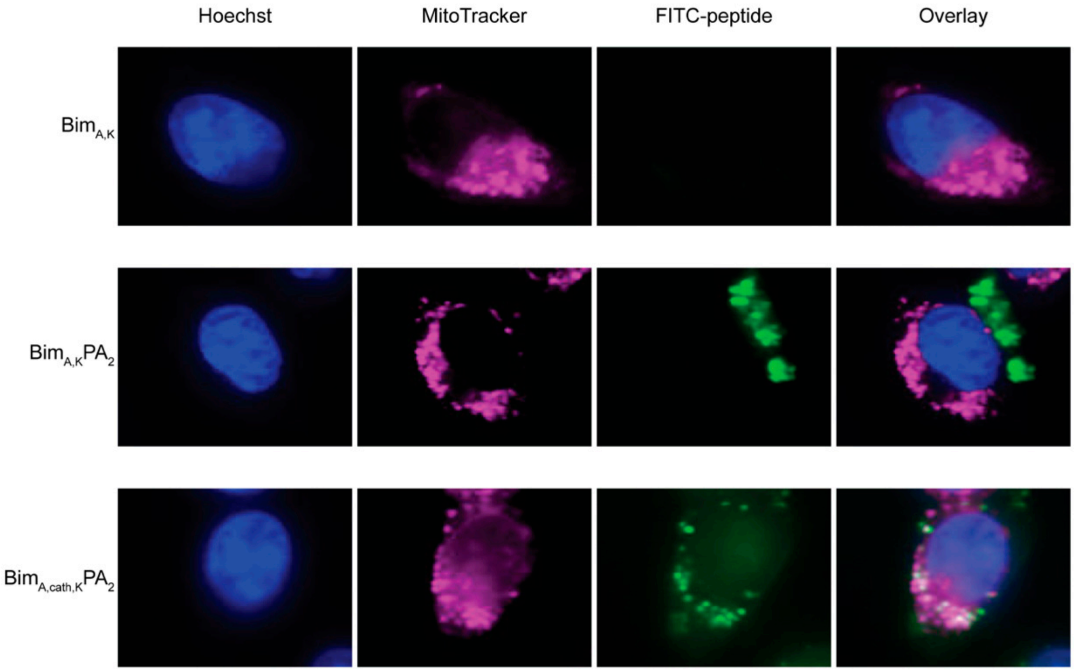

Figure S3. Live cell confocal microscopy of HeLa cells treated with FITC-labeled $BIM_{A,K}$ peptide, $BIM_{A,K}PA_2$, or $BIM_{A,cath,K}PA_2$ for 2 h followed by washing. Only $BIM_{A,cath,K}PA_2$ enabled FITC-peptide co-localization with MitoTracker-labeled mitochondria. Original magnification, ×100.

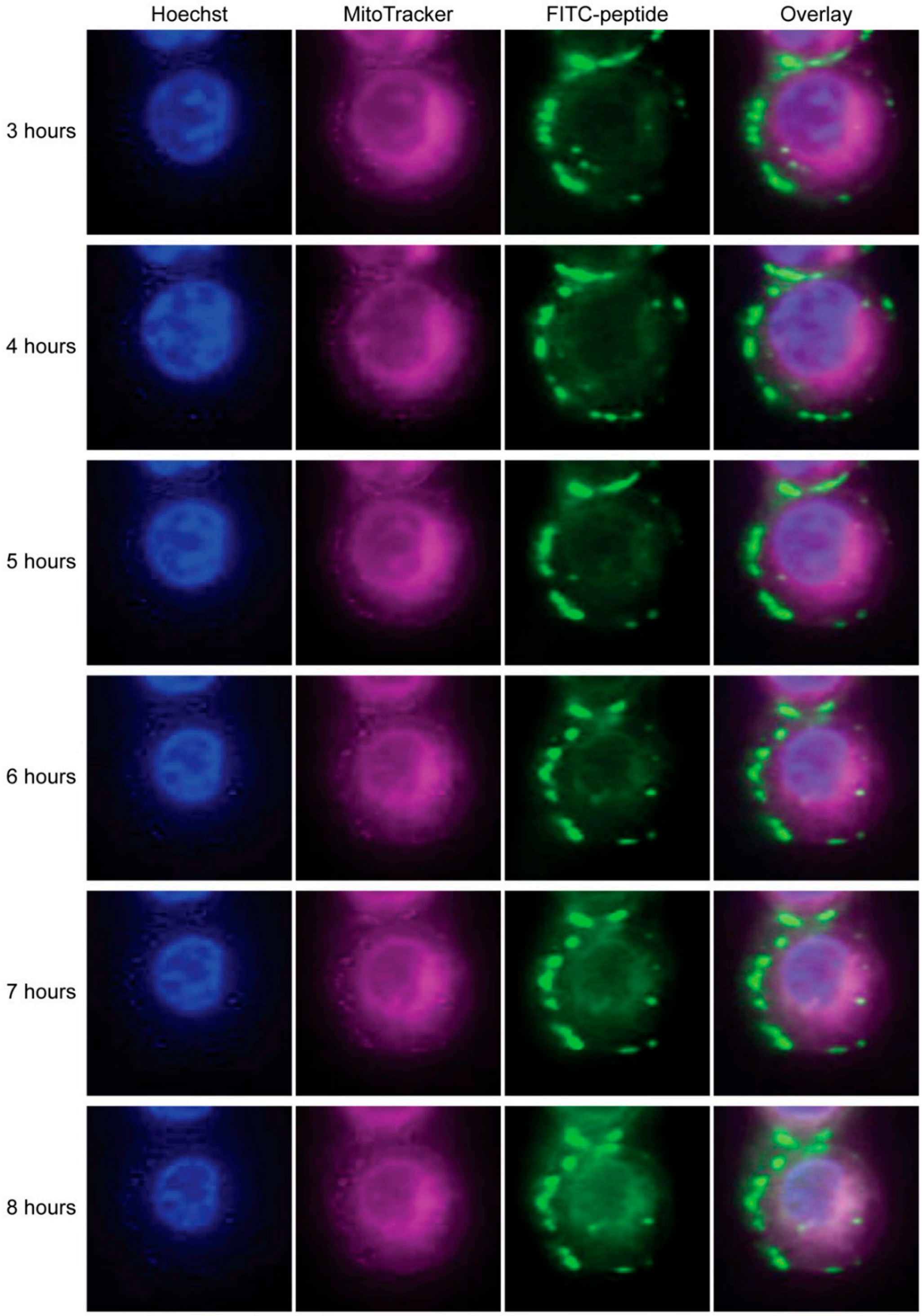

Figure S4. Time-lapse, live cell confocal microscopy of HeLa cells treated with FITC-labeled $BIM_{A,cath,K}PA_2$. Cells were treated with 10 μM FITC-$BIM_{A,cath,K}PA_2$ for 2 h before being washed, stained, and imaged. FITC signal was first visible near the edges of the cell, and over 8 h, became diffusely fluorescent and co-localized with MitoTracker-labeled mitochondria. Original magnification, ×100.

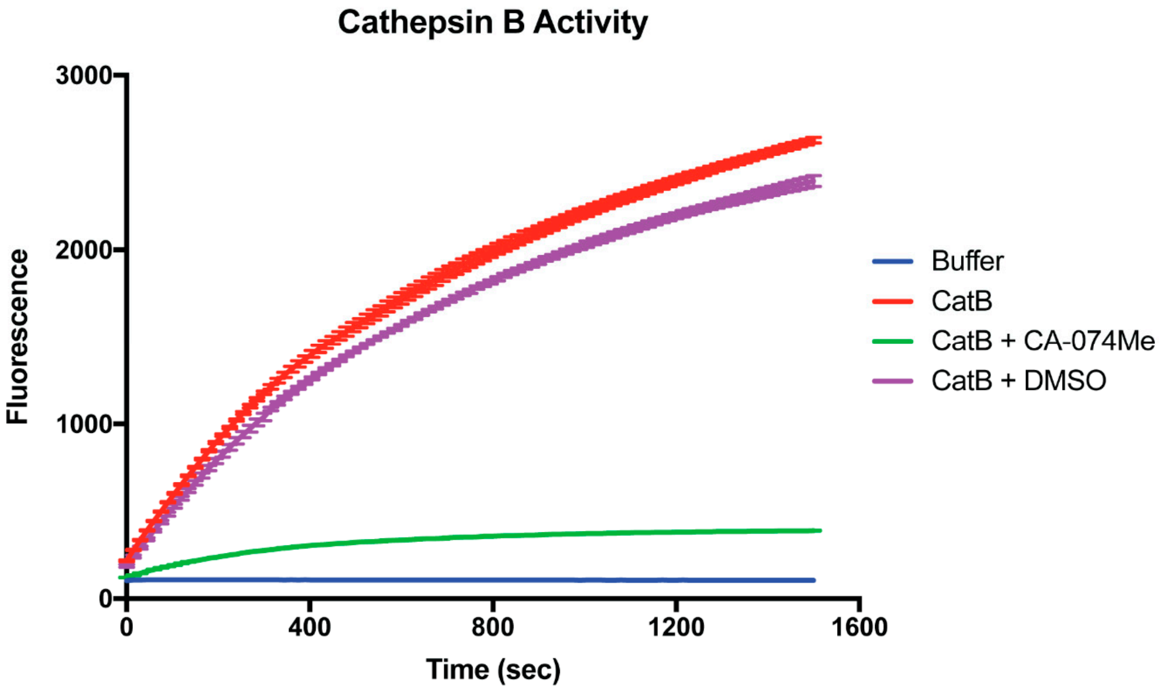

Figure S5. The cathepsin B inhibitor CA-074Me efficiently inhibits recombinant cathepsin B activity in vitro. Recombinant cathepsin B was added to a linker substrate that becomes fluorescent following cathepsin cleavage. The reaction was co-incubated with either CA-074Me or DMSO vehicle control.

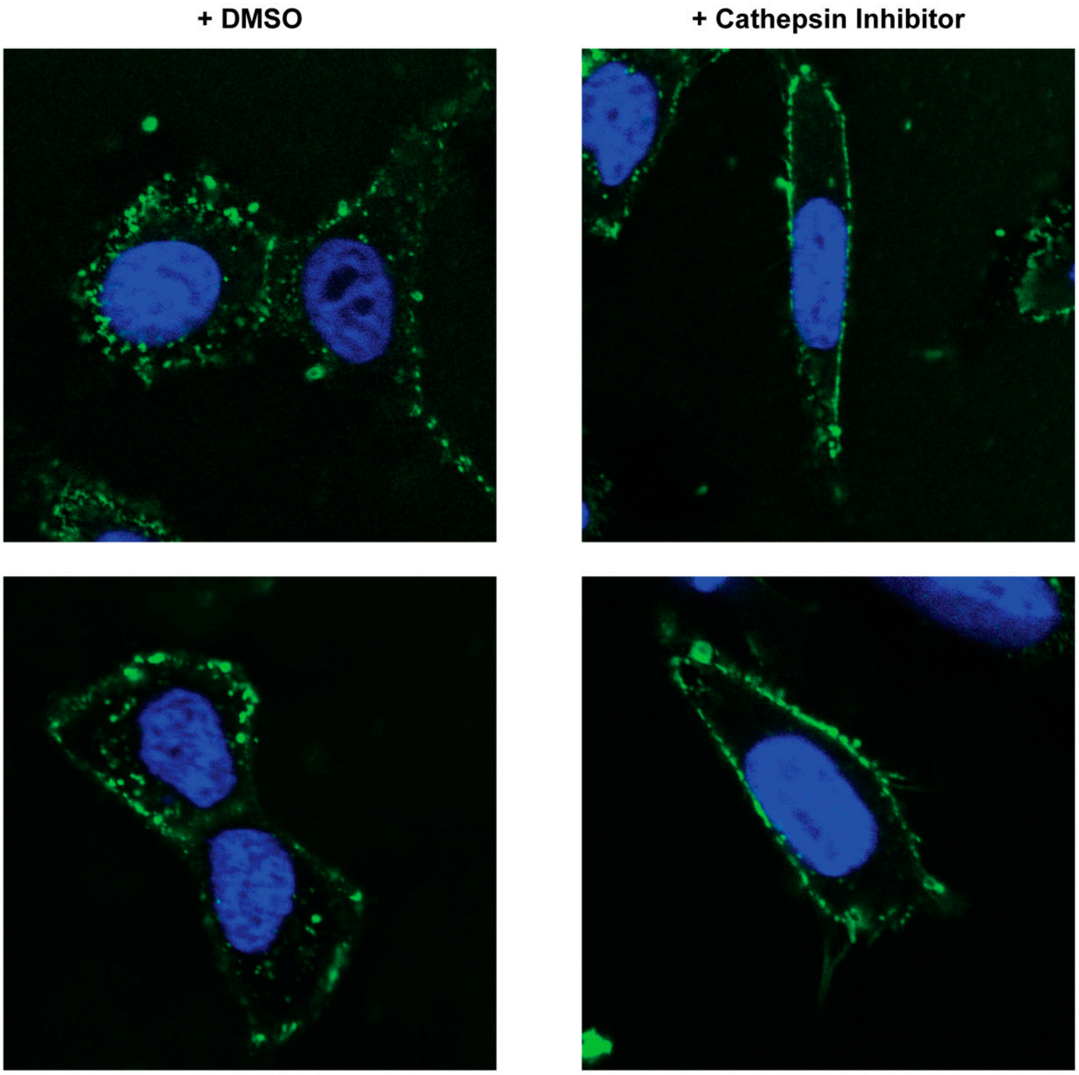

Figure S6. The cathepsin inhibitor, CA-074Me, inhibits $BIM_{A,cath,K}PA_2$'s cellular uptake. MEFs were pre-incubated with either 5 μM CA-074Me or 0.1% (v/v) DMSO control in complete media for 1 h. They were then washed and treated with 10 μM FITC-$BIM_{A,cath,K}PA_2$ for 1 h before washing, fixation, staining with Hoechst, and confocal imaging.

© 2019 by the authors. Submitted for possible open access publication under the terms and conditions of the Creative Commons Attribution (CC BY) license (http://creativecommons.org/licenses/by/4.0/).
